# Retraction: The Immunoregulation Effect of Alpha 1-Antitrypsin Prolong β-Cell Survival after Transplantation

**DOI:** 10.1371/journal.pone.0200182

**Published:** 2018-06-28

**Authors:** The PLOS ONE Editors

After publication of this article [1], concerns were raised about similarities between images shown in Figures 3 and 5:

- Figures 3A and 3B appear highly similar in quadrants P1, P2, P3, and select areas of P4.
- Within Figures 3C and 3F, there are areas that appear to be duplicated within the plots in R2 and R4 quadrants.
- Similarities were noted between Figures 3D and 3E (quadrants P1, P3, P4), and between Figures 3D, 3E, and 3G (quadrants P2, P2, R2, respectively).
- Similarities between Figures 5C and 5D suggest they may be derived from the same image, although they reportedly show kidney capsule samples isolated at different timepoints (14d, 28d post-transplantation).

In response to queries about these concerns, the corresponding author requested retraction, apologized for the figure issues, and indicated that the original data underlying these results are no longer available.

In light of concerns about the integrity of these data, the *PLOS ONE* Editors retract the article.

SYZ, FRL agreed with the retraction. YW, HJY, HQ, CYD did not respond.
